# Improving Performance of Breast Lesion Classification Using a ResNet50 Model Optimized with a Novel Attention Mechanism

**DOI:** 10.3390/tomography8050200

**Published:** 2022-09-28

**Authors:** Warid Islam, Meredith Jones, Rowzat Faiz, Negar Sadeghipour, Yuchen Qiu, Bin Zheng

**Affiliations:** 1 School of Electrical & Computer Engineering, University of Oklahoma, Norman, OK 73019, USA; 2 Stephenson School of Biomedical Engineering, University of Oklahoma, Norman, OK 73019, USA

**Keywords:** computer-aided diagnosis (CAD) scheme of mammograms, convolutional block attention module (CBAM), residual network (ResNet), breast lesion classification

## Abstract

**Background:** The accurate classification between malignant and benign breast lesions detected on mammograms is a crucial but difficult challenge for reducing false-positive recall rates and improving the efficacy of breast cancer screening. **Objective:** This study aims to optimize a new deep transfer learning model by implementing a novel attention mechanism in order to improve the accuracy of breast lesion classification. **Methods:** ResNet50 is selected as the base model to develop a new deep transfer learning model. To enhance the accuracy of breast lesion classification, we propose adding a convolutional block attention module (CBAM) to the standard ResNet50 model and optimizing a new model for this task. We assembled a large dataset with 4280 mammograms depicting suspicious soft-tissue mass-type lesions. A region of interest (ROI) is extracted from each image based on lesion center. Among them, 2480 and 1800 ROIs depict verified benign and malignant lesions, respectively. The image dataset is randomly split into two subsets with a ratio of 9:1 five times to train and test two ResNet50 models with and without using CBAM. **Results:** Using the area under ROC curve (AUC) as an evaluation index, the new CBAM-based ResNet50 model yields AUC = 0.866 ± 0.015, which is significantly higher than that obtained by the standard ResNet50 model (AUC = 0.772 ± 0.008) (*p* < 0.01). **Conclusion:** This study demonstrates that although deep transfer learning technology attracted broad research interest in medical-imaging informatic fields, adding a new attention mechanism to optimize deep transfer learning models for specific application tasks can play an important role in further improving model performances.

## 1. Introduction

Breast cancer is among the leading cause of death among women around the world. The early detection and diagnosis of breast cancer can play an important role in reducing the mortality rate of breast cancer patients due to early and proper treatments. Thus, breast cancer screening has been widely promoted and used in the last several decades. Currently, digital mammography is still the primary imaging screening tool used in breast cancer screening due to its advantages of having low costs, wide accessibility, and short screening times. However, the efficacy of population-based screening mammography remains controversial (particularly due to high false-positive recalls) [1]. As a result, the majority of breast lesion biopsy results are benign. This not only wastes healthcare resources but also creates anxieties among women with false-positive detections or benign biopsies, which can induce long-term psychosocial consequences among these women [2] and render them less likely to continue participating in breast cancer screening [3]. Therefore, there is substantial research interest aiming to develop novel quantitative image markers or decision-making supporting tools that can effectively assist radiologists in making more accurate diagnostic decision to classify suspicious or worrisome breast lesions detected on mammograms.

In the last two decades, many researchers developed and tested a variety of computer-aided diagnosis (CAD) schemes to classify malignant and benign breast lesions [4]. However, the performance of these CAD schemes heavily depends on breast lesion segmentation and the selection of optimal “handcrafted” image features, which remain challenging technical issues in CAD development [5,6]. Thus, the performance and scientific rigor of the conventional CAD schemes remain lower. Recently, due to rapid developments in deep learning technology, training and applying deep learning models to develop new CAD schemes of medical images including mammograms to classify malignant and benign breast lesions have attracted broad research interest [7,8]. Using a deep learning method can typically avoid lesion segmentation processes and use model-generated automated image features to build and train the final lesion classifier. Specifically, by using a relatively large and diverse image dataset, a deep learning model can learn intricate patterns from large dimensionally raw data with no or little human guidance [9,10].

Among many different deep learning models, the convolutional neural network (CNN) is the most popular deep learning architecture [11]. Because of its relatively simple but robust characteristics [12], CNN-based deep learning models have been widely applied to develop CAD schemes of medical images [13,14,15,16,17]. However, one of the major drawbacks of the convolutional neural network is that it requires large amounts of data for training, which is sometimes difficult to obtain in medical imaging fields. Thus, transfer learning has recently become a popular strategy among researchers to address this issue [18,19]. The current standard of transfer learning is to select and use an existing model (i.e., an AlexNet model [20]) that was originally trained using a large natural image dataset such as ImageNet [21] and then to finetune the model using limited medical imaging data. Despite the advantages of CNN, one of the major difficulties or challenges of using CNNs is how to optimally train or finetune the model due to the problem of vanishing gradients, which may make the training process difficult in identifying the “best” or optimal converging result. One solution that has been used in previous studies is to place an auxiliary loss in an intermediate layer as additional monitoring, but it does not solve the problem completely.

Thus, many modified or advanced deep learning models have recently been developed and tested. Previous studies have compared many different deep learning models and found that ResNet50 model was the best architecture framework for the image classification task with the highest accuracy and efficiency to train [22,23]. As a result, recently, a ResNet50 model has also been used to build a new transfer learning model or CAD scheme of mammograms to classify breast lesions [24]. On the other hand, a new technology, namely, attention mechanisms, has also become a popular method used in image analysis and demonstrates their unique capability and advantages in boosting the representational strength by emphasizing crucial details and squelching superfluous ones [25], which have been applied in computer vision systems of object recognition [26]. In particular, a convolutional block attention module (CBAM) is designed to highlight significant characteristics along the two principal dimensions, namely, channel and spatial attention axes. Each branch learns “what” and “where” to attend in the channel and spatial axes, respectively, by applying channel and spatial attention modules in order, which can more effectively identify and distinguish different image patterns. However, despite the advantages, to the best of our knowledge, CBAM has not been used to help develop deep transfer learning models, particularly for the task of classifying medical images, including classifying breast lesions using mammograms.

Therefore, based on the literature review, we propose a hypothesis as follows: Adding an optimal attention mechanism (i.e., a CBAM) to the finetuning process of ResNet50 model can combine the advantages of two technologies, which will have significant advantages in developing a new CAD scheme and further improve the CAD performance of breast lesion classification using mammograms. The objective of this study is to test or verify the above hypothesis. The details of this study are presented in the following sections of this paper.

## 2. Materials and Methods

### 2.1. Image Dataset

In this study, we assembled a retrospective image dataset from an existing mammography database previously collected in our medical imaging laboratory, which has been used to develop several CAD schemes reported in our previous research papers (i.e., [27,28,29]). The dataset used in this study includes 4280 mammograms. Each mammogram contains a suspicious soft-tissue mass-type breast lesion that was detected by a radiologist during initial image reading and diagnosis in mammography screening. All suspicious lesions were recalled and had biopsy tests. Based on the confirmed pathology examination results, 2480 images include benign lesions, while the rest of the 1800 images depict malignant lesions in this image dataset.

**Figure 1:**
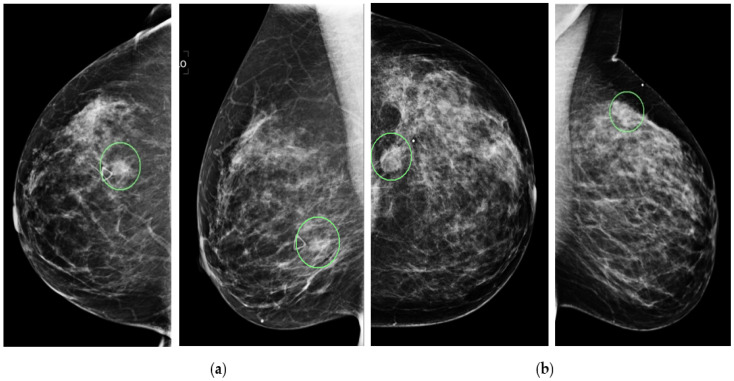
Illustration of four mammograms of CC and MLO views of two patients in which two suspicious mass type lesions are detected, as circled by green rings. Two images of one patient depict a benign lesion (**a**) and two images of another patient depict a malignant lesion (**b**).

All detected breast lesions are also previously annotated by the radiologists. Figure 1 shows four example mammograms from both the craniocaudal (CC) and mediolateral oblique (MLO) views acquired from two patients. In each image, the lesion’s center location is annotated with a green circle, as shown in the figure. Next, in this study, we extract a region of interest (ROI) with a fixed size of 128 × 128 pixels from each image. The annotated lesion center and the extracted ROI center overlapped. If part of the ROI is beyond the boundary of the original mammogram (i.e., a lesion that is detected close to the edge of the image as shown in Figure 1b), a zero-pad correction method is applied. The same size ROI or patch has been affectively used in our previous CAD studies (i.e., [24]). This relatively large and diverse image dataset is then used in this study to develop and test our proposed new deep transfer learning ResNet50 model implemented with a novel convolutional block attention module (CBAM), as discussed below.

### 2.2. A New Integrated Transfer Learning Model

#### 2.2.1. A ResNet 50 Model

In brief, the basic concept of ResNet is to introduce an identity shortcut relation that skips one or more layers [30]. It employs a technique known as skip connections, which link skips connections on two to three layers containing ReLU and batch normalization among the architectures. It allows the network to match the residual mapping rather than enabling the network to learn the underlying mapping. For example, let us consider H(x) as an underlying mapping to be fit by a few stacked layers, with “x” denoting the inputs to the first of these layers. If several nonlinear layers are thought to be able to asymptotically approximate complex functions, this is the same as thinking they can asymptotically approximate a residual function defined as F(x): = H(x) − x. The original function thus becomes F(x) + x. Although it should be possible for both forms to asymptotically approximate the desired functions (as was originally envisioned), the learning curves for each form may vary.

**Figure 2:**
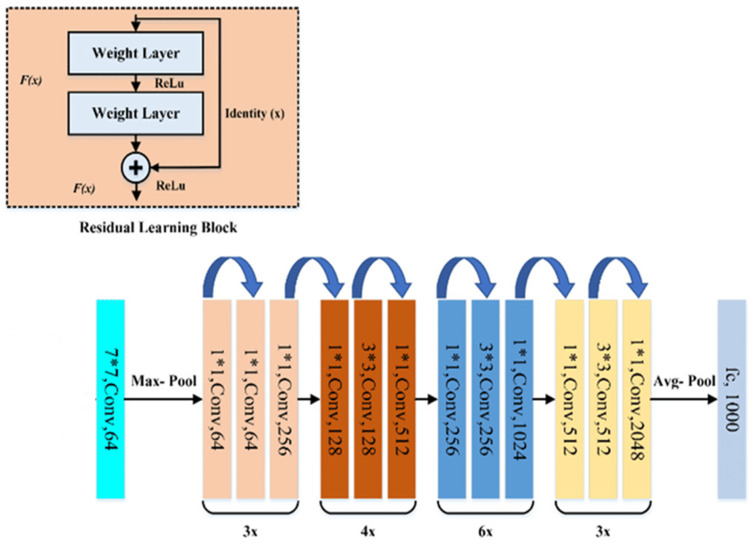
Illustration of a ResNet50 architecture or model.

The benefit of using the skip link is that any layer that degrades the design performance will be skipped by regularization. The training of very deep neural networks is therefore not hampered by vanishing gradients as conventional CNN models. The parametric gates are used in these skip connections, much like they are in long short-term memory (LSTM) networks. These gates control the quantity of data that moves across the skip connection. The gradient vanishing and feature map vanishing issues are addressed by ResNet when training an excessive number of deep CNN. Identity links between non-adjacent layers have no impact on the ideal mapping as the application task wants to produce, which is why the ResNet works. As a result of the gradients having access to an additional shortcut channel thanks to the identity connection, back propagation is more fluid. In summary, ResNet50 is a popular deep residual network that achieves a good balance between the depth of network and training efficiency. A typical ResNet50 architecture is shown in Figure 2, which is originally trained using more than a million images collected from the ImageNet database (*ImageNet*. http://www.image-net.org) (accessed on 1 August 2022).

#### 2.2.2. Channel Attention Module

**Figure 3:**
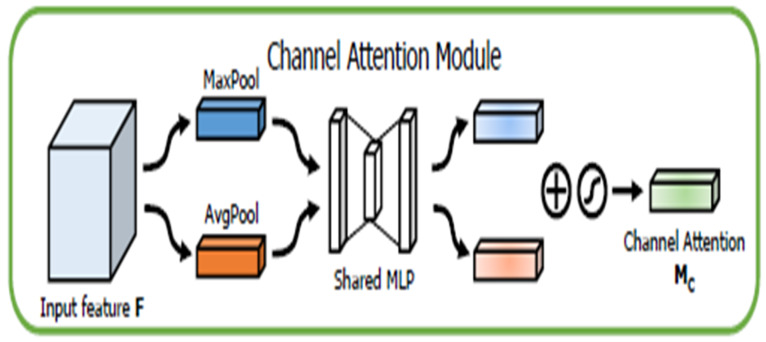
Illustration of a channel attention module.

Several different attention modules have also been investigated and applied in deep learning field to help improve deep learning model-training efficacy and results. Among them, the channel attention module focuses on “what” is meaningful given an input image as each channel of a feature map is considered as a feature detector [31]. Figure 3 provides a schematic of the process. To efficiently compute the channel attention, the spatial dimension of the input feature map is squeezed. The max-pooled and average pooled features are employed together to infer finer channel-wise attention. Both feature descriptors, $F_{avg}^{c}$ and $F_{max}^{c}$, are then forwarded to a shared network to produce the channel attention map: $M_{c}$ ∈ ${\mathbb{R}}^{C\times 1\times 1}$. The shared network is composed of a multi-layer perceptron (MLP) with one hidden layer. The output feature vectors are merged using element-wise summation, as shown in the equation below:

(1) $$M_{c}(F)=\sigma (\mathrm{MLP}(\mathrm{AvgPool}(F))+\mathrm{MLP}(\mathrm{MaxPool}(F)))\;=\sigma \;(W_{1}(W_{0}(F_{avg}^{c}))+W_{1}(W_{0}(F_{max}^{c})))\;$$

where σ denotes the sigmoid function, $W_{0}$ ∈ ${\mathbb{R}}^{\left(\frac{C}{r}\right)\times C}$, and $W_{1}$ ∈ ${\mathbb{R}}^{C\times \left(\frac{C}{r}\right)}$. MLP weights, $W_{0}$ and $W_{1}$, are shared for both inputs, and the ReLU activation function is followed by $W_{0}.$

#### 2.2.3. Spatial Attention Module

**Figure 4:**
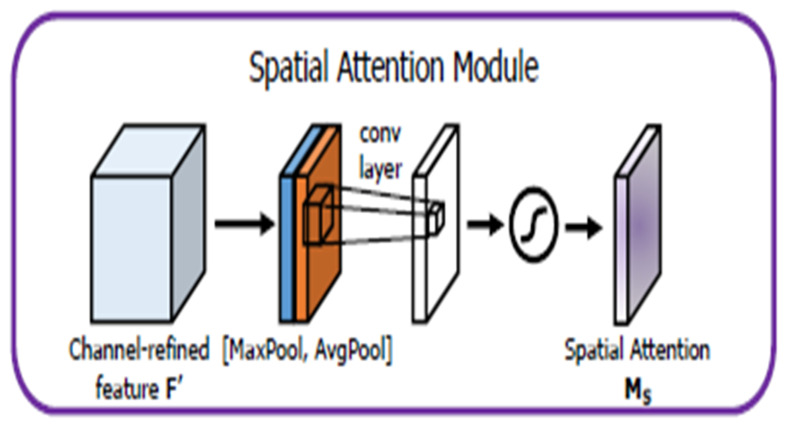
Illustration of a spatial attention module.

Another popular attention module is a spatial attention module that is generated by utilizing the inter-spatial relationship of features. This module emphasizes on “where” an informative part is, which is complementary to the channel attention module. At first, average-pooling and max-pooling operations are applied along the channel’s axis. Applications of pooling techniques along the channel axis demonstrated success in emphasizing informative sections [32]. Then, they merge to generate an efficient feature descriptor. A convolution layer is applied on the merged feature descriptor to generate a spatial attention map $M_{s}$(F) ∈ ${\mathbb{R}}^{H\times W}$, which encodes the location to focus on or suppress. The detail of the spatial attention module is illustrated in Figure 4:

(2) $$M_{s}\left(F)=\sigma (f^{7\times 7}([\mathrm{AvgPool}(F);\;\mathrm{MaxPool}(F)])\right)\;=\sigma \;(f^{7\times 7}([F_{avg}^{s};F_{max}^{s}]))\;$$

where σ denotes the sigmoid function, and $f^{7\times 7}$ represents a convolution operation with a filter size of 7 × 7.

#### 2.2.4. Convolution Block Attention Module

**Figure 5:**
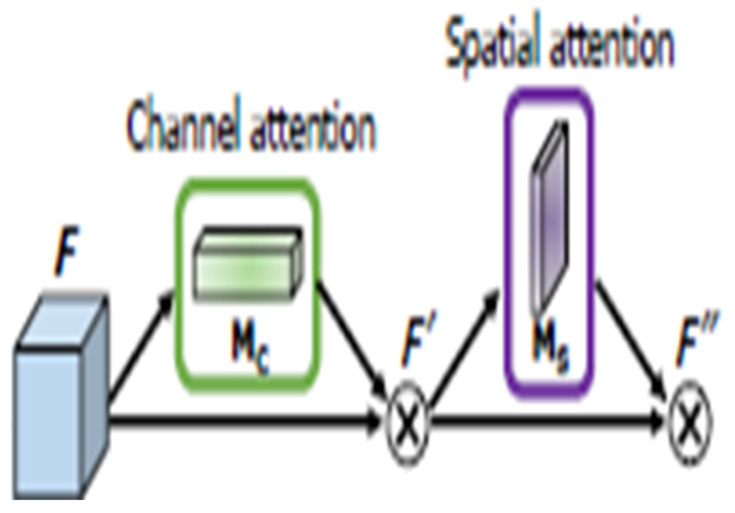
Illustration of a convolutional block attention module (CBAM).

Due to the complementary characteristics between the channel attention module and the spatial attention module, a new attention module was developed to sequentially combine these two modules in an attempt to take advantages of these two attention modules. This new module is named as the convolution block attention module (CBAM). The overall attention process of CBAM [33] can be summarized as follows:

(3) $$F^{\prime }=M_{c}(F)\;⊗\;F$$

(4) $$\;F^{\prime \prime }=M_{s}\left(F^{\prime }\right)\;⊗F^{\prime }\;$$

where ⊗  denotes element-wise multiplication. *F″* is the final refined module output. The basic architecture of the CBAM is illustrated in Figure 5. Due to its combined capability or advantages, CBAM has recently been attracting research interest in medical imaging field [34,35]. However, CBAM has not been explored in helping develop deep learning models for breast lesion classification. In this study, we propose to add CBAM to the ResNet50 fine-tuning process in order to improve transfer learning efficacies and breast lesion classification performance using the CAD scheme implemented with the ResNet50 model.

### 2.3. Transfer Learning Process

**Figure 6:**
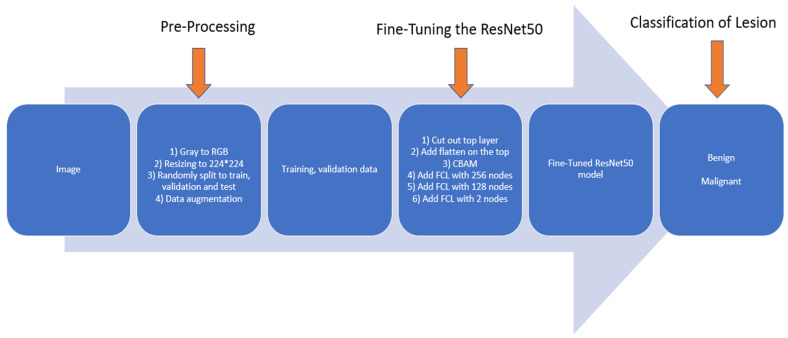
Schematic of our proposed CBAM-based ResNet50-model-based transfer learning process.

In the original or standard ResNet50 model, all network connection weights are pre-trained using a large and diverse ImageNet database to recognize or classify 1000 different objects, which thus has 1000 nodes in the last classification layer. In this study, we use the pre-trained ResNet50 model as a base to build two transfer learning models or breast lesion classifiers with and without adding CBAM in the finetuning process. Two new models only include two output nodes to represent benign and malignant lesions. For this purpose, we take following steps to conduct the transfer learning process to build breast lesion classifiers, as shown in Figure 6.

First, we apply several image pre-processing steps to optimally use mammograms as input images of the model. The steps include the following: (1) gray to RGB: the original grayscale mammogram is simply copied into all three (RGB) input channels of the ResNet50 model. (2) Resizing to 224 × 224: Images in all three channels are resized to 224 × 224 pixels using the standard mathematics interpolation method to fit the default image size of the ResNet50 model. (3) Randomly split the original image dataset into to train, validation, and test subsets (which will further be discussed in Section 2.4). (4) Data augmentation: The standard augmentation method is applied to increase the size of training samples and diversity in lesion orientations.

Second, to optimally control training iteration epochs in order to reduce overtraining bias, we divide the training dataset into two subgroups to be used for model training and validation using a ratio of 9:1. We will use validation data and validation results to control model training or fine-tuning processes, such as the number of training epochs.

Third, we modify the original architecture of the ResNet50 model, which is shown in Figure 2. Specifically, the original architecture of ResNet50 remains unchanged (i.e., the weights and biases remain the same) until the last or top Fully Connection Layer (FCL), which are removed. Specifically, the last layer in the original architecture as shown in Figure 2 is removed at the start of the model’s finetuning process. A new flatten layer is added to the model. We also integrate the CBAM to ResNet50 after adding the new flattened layer. Next, we add two FCLs with 256 and 128 nodes. In these two convolution layers, Rectified Linear Unit (ReLU) [36] is used as the activation function. In the final step of this finetuning process, we add the last classification layer, which uses Softmax as the activation function to fulfill a new two-class breast lesion classification task.

Fourth, in order to train or finetune the new transfer learning model, the complete model is compiled with the Adam [37] optimizer with a batch size of 32, the max epoch is limited to 100, and the initial learning rate is chosen as $2\times 10^{-5},$ which is smaller than the learning rate of ResNet50 used on ImageNet due to the small dataset of mammograms. The small learning rate preserves valuable parameters as much as possible by avoiding dramatic changes on the pre-trained parameters and lets the model better learn the input images more minutely.

### 2.4. Model Training and Testing

After determining the architecture of our modified transfer learning ResNet50 model, we apply following steps to train and test two classifiers based on transferred ResNet50 model with and without adding CBAM in the model fine-tuning process. Specifically, we randomly split the entire image dataset of 4280 images into 3 independent subsets of training, validation, and testing. Overall, 10% of images (428) were assigned to testing subset. In the rest of the dataset, 90% of the images (3467) formed a training subset, while 10% of the images (385) were assigned to the validation subset. To maintain the same image partition ratios for the two classes of benign and malignant breast lesions, the case partition of the assignment was performed on images of both benign and malignant lesion classes independently using the same case-splitting ratios.

On the training data subset, we applied shearing, zoom, rotation, width and height shift, and horizontal flip as augmentation techniques that have been commonly used in training deep learning models, aiming to increase the size of training samples [38]. The values of these parameters are shown in Table 1.

Next, multiple iteration or epochs are applied to train the CBAM-based ResNet50 model. The model is first trained or finetuned using the data in the training subset and then tested using the validation subset. To close the performance gap between training and validation datasets, the optimizer tries to push the architecture to learn more data during training. To control overfitting and maintain training efficiencies, we limit model training epochs to 100 in the experiment. During these 100 epochs, an optimally trained model based on validation dataset result is saved and then the trained model is tested “as is” using the data in the independent testing subset, which does not involve in the model training and validation process.

**Figure 7:**
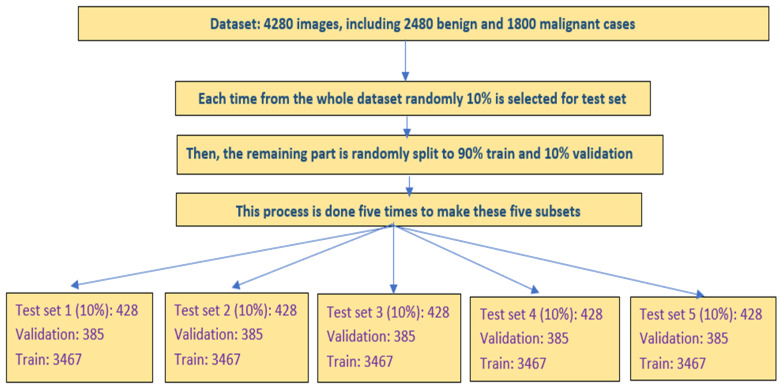
Schematic diagram of model training, validation, and testing phase.

Additionally, in order to evaluate the robustness or rigor of the above training, validation and testing process, we repeat this model training and testing process five times by randomly dividing all images into 3 subsets of training, validation, and testing five times using the same image partition ratios in order to reduce the risk of potential bias in image data partitions [39]. Specifically, the image assigned to the validation and testing subsets is entirely different for these five instances of image partitions. As a result, five sets of the trained deep transfer learning models are validated and tested with utterly unique testing scenarios. Therefore, the total number of testing images increases to 2140 (428 × 5). Figure 7 shows a schematic diagram of the training, validation, and testing phase of this experiment.

### 2.5. Performance Evaluation

We apply the following two statistical data analysis methods to evaluate and compare breast lesion classification performances or accuracy levels of two transfer learning ResNet50 model with and without adding CBAM. First, we use a receiver operating characteristic (ROC) type data analysis method and the area under the ROC curve (AUC) as an evaluation index. Second, based on model-generated probability scores between two output/classification nodes that represent malignant and benign lesions, a testing image is assigned to one class of either malignant or benign lesion, based upon which the probability score is greater between two output nodes of the model. Then, a confusion matrix is generated for each testing subset. Based on the confusion matrix, several additional evaluation indices are computed, which include the overall classification accuracy, sensitivity, specificity, and F1 score computed as follows to evaluate model performances used in a binary or practical decision environment:

(5) $$Accuracy=\;\frac{TM+TB}{All\;images}\;$$

(6) $$Sensitivity=\;\frac{TM}{TM+FB}$$

(7) $$Specificity=\;\frac{TB}{TB+FM}$$

(8) $$F1=2\times \frac{TM^{2}}{TM\times \left(2\times TM+FM+FB\right)}$$

where *TM* and *TB* represent the number of correctly classified true malignant lesion and true benign lesions, respectively, while *FM* and *FB* represent misclassified malignant and benign lesions, respectively.

## 3. Results

First, the experimental results show that when applying five transfer learning ResNet50 models optimized with CBAM to five independent testing datasets, the computed AUC values are 0.86, 0.87, 0.89, 0.86, and 0.85, which results in a mean AUC value of 0.866 and a standard deviation of 0.015. When testing five standard transfer learning ResNet50 without adding CBAM, AUC values are 0.77, 0.78, 0.79, 0.79, and 0.78 for the five testing datasets, in which the mean AUC value is 0.780 and the standard deviation is 0.008. By comparing AUC values generated by two models, the ResNet50 model optimized with CBAM yields a significantly higher mean AUC value (*p* < 0.01).

**Figure 8:**
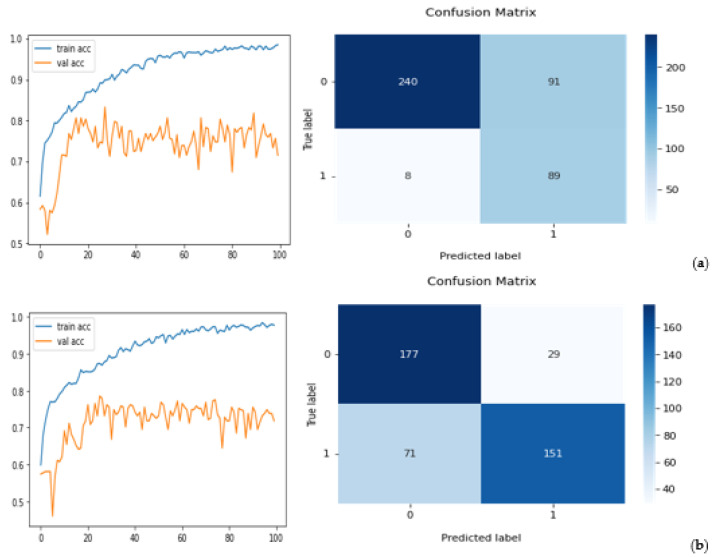

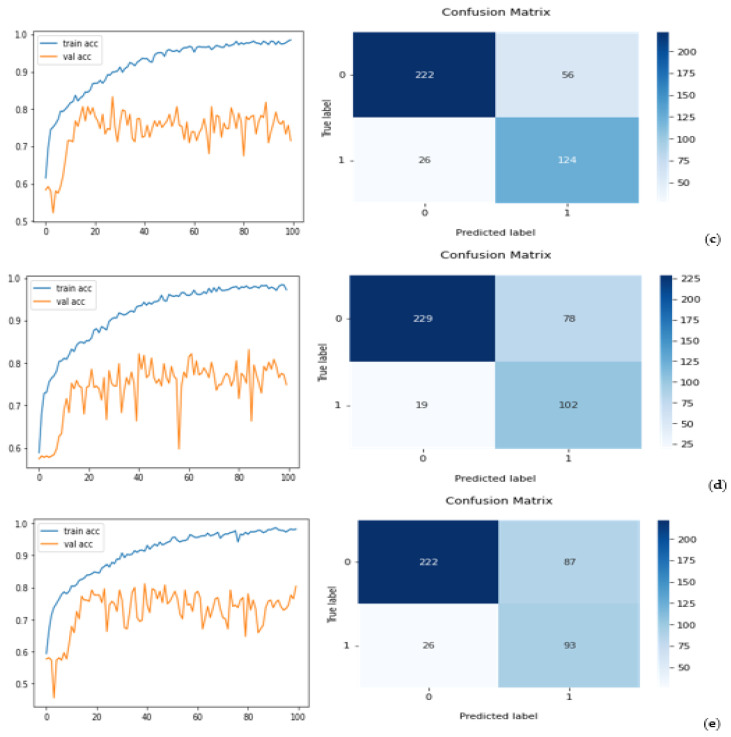
Left column show five sets of performance curves (**a**–**e**) of the ResNet50 model optimized with CBAM, applied to the training and validation subsets for five experiments in 100 training epochs. The horizontal axis shows the number of epochs, and the vertical axis shows the accuracy. The right column shows five confusion matrices of the corresponding testing results. In labeling axes of each confusion matrix, “1” represents a malignant lesion class, while “0” represents a benign lesion class.

Second, Figure 8 demonstrates classification performance trends and confusion matrices generated by five repeated experiments. Specifically, the left columns of Figure 8 present five trend curves of training (or fine-tuning) and validation accuracy of the ResNet50 model optimized with CBAM using different training and validation image data subsets. These curves show that the training and validation accuracy increases as the number of learning iterations (epochs) increases initially. However, after epoch 20, the models do not show the trend of a continuous improvement in lesion classification, while overfitting risk increased. Thus, we chose epochs = 20 to train and build all five models to prevent the risk of model overfitting. Then, by applying the trained models to the corresponding independent testing subsets, five confusion matrices that are computed based on the lesion classification of testing datasets using the ResNet50 model optimized with CBAM are displayed in the right columns of Figure 8.

Third, from these five confusion matrices, the computed lesion classification accuracy values are 0.78, 0.76, 0.79, 0.76, and 0.78, which indicates that the mean accuracy value of lesion classification is 0.774 ± 0.019. Additionally, the mean sensitivity and specificity values are 0.77 ± 0.05 and 0.81 ± 0.06, respectively. Table 2 lists and compares five sets of the lesion classification index data computed based on lesion classification results between using two transfer learning ResNet50 models with and without adding CBAM in the model’s training process. The results show that using ResNet50 with CBAM achieves a higher performance, as evauated by all five indicies reported in Table 2.

**Figure 9:**
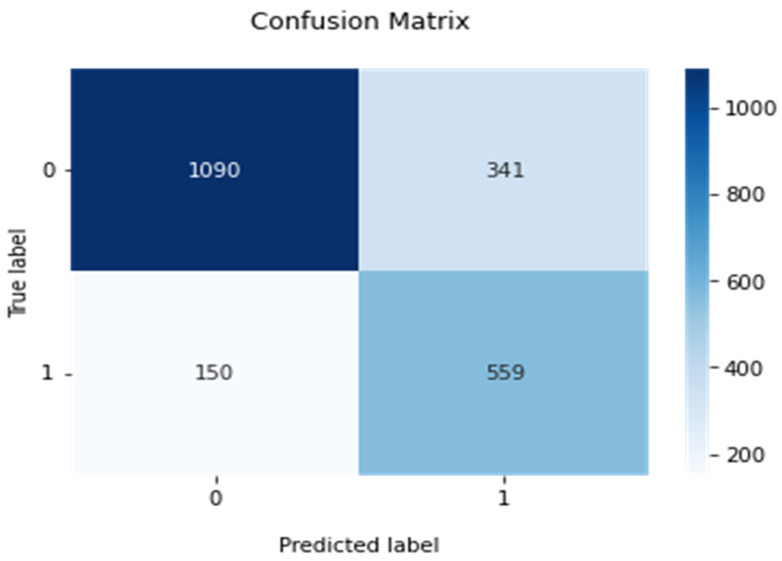
A combined confusion matrix of applying five trained models to five independent testing data subsets with total 2140 cases of the ResNet50 model optimized with CBAM (representing lesion classification result on the pooling testing data). In labeling axes of the confusion matrix, “1” represents a malignant lesion class, while “0” represents a benign lesion class.

Fourth, Figure 9 shows another confusion matrix by combining or pooling all 2140 testing images in all five independent training and testing experiments. The computed overall accuracy of the pooling testing images is 0.77 (1649/2140), while the sensitivity and specificity are 0.77 and 0.79, respectively. The performance indices are quite comparable to the results of mean index values, as reported in Table 2, using five smaller testing datasets, which also demonstrates the robustness of the ResNet50 model finetuned with CBAM for this breast lesion classification task.

## 4. Discussion

Deep learning technology and a variety of models with different architectures have recently been widely used to develop new CAD schemes for medical images, including classifying breast lesions detected on mammograms. However, due to the use of small training image datasets and/or possible bias in image partitions, the performance of the deep learning models of medical images varies greatly with lower scientific rigor. As a result, most deep learning models have pitfalls or bias and are not acceptable in clinical practice [40]. In this study, we investigate a novel approach to develop a new CAD scheme in order to classify benign and malignant breast lesions detected on mammograms. Our study has several unique characteristics and study results have also demonstrated several advantages of our new approach.

First, although a ResNet50 model has significant advantages over many other existing deep learning models in performing object recognition or classification tasks, directly adopting the ResNet50 model to develop a new transfer learning model to classify breast lesion detected on mammograms may not be an optimal or accurate approach because breast lesions are more subtle and heterogenous than many other targeted objects. To address this challenge and improve model performances, we investigate and test a unique approach by adding a novel attention module, namely, a convolutional block attention module (CBAM), to help finetune and optimize the ResNet50 model to classify breast lesions. Our study results demonstrate that the ResNet50 model optimized using CBAM yields significantly higher breast lesion classification performance (AUC value) than the standard ResNet50 model without using CBAM in its finetuning process. This indicates the importance of the research effort to continuously search for novel and more effective model finetuning and optimization methods in developing deep transfer learning model-based CAD schemes with respect to medical images.

Second, although several different attention modules are currently available for selection and applied to help finetune and optimize deep transfer learning models, this study also demonstrates that using CBAM has the advantage of combining complementary learning approaches to achieve higher performance. Specifically, CBAM is a combination of two popular attention modules, namely, the Channel Attention Module (CAM) and Spatial Attention Module (SAM), as shown in Figure 6. In order to investigate and/or demonstrate the advantages of using CBAM over the use of these two attention modules independently, we also compare the lesion classification performance of three ResNet50 models optimized by separately adding these three attention modules. Table 3 shows and compares the breast lesion classification performance of three ResNet50 models optimized by adding three attention modules. The comparison data indicate that the ResNet50 model optimized by adding CBAM achieves the highest breast lesion classification performance evaluated using all five evaluation indices.

Third, this study uses a relatively large and diverse image dataset with 4280 mammograms or lesion regions. In particular, 3467 images were randomly selected to finetune ResNet50 models. Although the size of this image dataset is much larger than most datasets used and reported in the previous studies of developing CAD schemes of breast lesion classification, we recognize the potential bias of image partitions in training, validation, and testing data subsets. Thus, in order to test the scientific rigor or robustness of the deep transfer learning ResNet50 model used for breast lesion classification, we randomly divided the dataset and repeated the experiment five times. As a result, five sets of ResNet50 models optimized or finetuned with and without adding CBAM were applied “as is” to five independent testing image datasets. No one image was selected and used twice as a testing image in these five repeated experiments. The data analysis results show small standard deviations (i.e., ≤0.02 or 2.3% for AUC values as shown in Table 2), which demonstrated that the finetuned transfer learning ResNet50 models are robust. This is encouraging and indicates that using a relatively large and diverse image dataset can play an important role in developing and training robust deep transfer learning models of mammograms. We believe that this concept of using larger training datasets and the approach to finetuning deep transfer learning models by adding or using an effective attention module are not only limited to this study with the task of breast lesion classification using mammograms, but the approaches are also applicable for developing and optimizing other deep transfer learning models for many other medical-imaging application tasks.

Despite the encouraging results or observations made in this study, we also recognize several limitations. First, the original ResNet50 model was designed to process color images with three (RGB) input channels. However, a mammogram is a gray-level image. In this study, we simply copy the same gray-level image into all three input channels of ResNet50 model, which may not be the optimal approach. Previous studies investigated different methods to create pseudo-color images by applying different image preprocessing algorithms and demonstrated the potential advantages of using pseudo-color images over only copying raw images into three input channels of the deep learning models [39,41]. Second, since the goal of this study is to investigate the feasibility and advantages of adding CBAM to help finetune the ResNet50 model, we directly used all model-generated automated image features to build the final lesion classifier, which may also not be an optimal approach because it misses a potentially important step of feature selection or the feature dimensionality reduction process as used in some of other previous studies [39,41]. Third, although this study uses a large and diverse image dataset, all images were acquired using one type of digital mammography machine in one medical center. The robustness of the ResNet50 models finetuned in this study needs to be tested and validated in future studies with more diverse image datasets acquired from different medical centers or hospitals. Thus, the above issues or limitations need to be further investigated in our future studies. However, we believe that this study is valid, and it successfully tests or verifies the hypothesis of this study discussed in the last paragraph of the Introduction section of this paper.

## 5. Conclusions

In this paper, we present a unique study that investigates the feasibility and advantages of developing a new CAD scheme based on a deep transfer learning ResNet50 model that is finetuned using a convolutional block attention module (CBAM) to classify malignant and benign breast lesions detected on mammograms. The study uses a relatively large and diverse image dataset with 4280 images or lesion regions. The data analysis’ results demonstrate that a transfer learning ResNet50 model finetuned by adding CBAM yields significantly higher lesion classification performance (AUC value) than a standard ResNet50 model without using CBAM. The robustness of the lesion classification results is also tested and demonstrated using five repeated experiments with independent testing image datasets. Thus, although deep transfer learning technology has attracted broad research interests in the medical-imaging informatics field, adding a new attention mechanism or other optimization methods to finetune deep transfer learning models for the specific application tasks can play an important role in further improving model performances.

## Figures and Tables

**Table 1:** Parameters used in data augmentation.

Shear Range	Zoom Range	Rotation Range	Width Shift Range	Height Shift Range	Horizontal Flip
0.2	0.2	40	0.2	0.2	True

**Table 2:** Performance comparison between two ResNet50 models with and without using CBAM.

Model	AUC	Accuracy	Sensitivity	Specificity	F1 Score
Standard ResNet50	0.77 ± 0.01	0.76 ± 0.03	0.76 ± 0.02	0.50 ± 0.09	0.67 ± 0.07
ResNet50 & CBAM	0.87 ± 0.02	0.77 ± 0.02	0.77 ± 0.05	0.79 ± 0.06	0.75 ± 0.05

**Table 3:** Comparison of breast lesion classification results of three transfer learning ResNet50 finetuned by adding three different attention modules.

Attention Module	AUC	Accuracy	Sensitivity	Specificity	F1 Score
CAM	0.82	0.74	0.74	0.71	0.74
SAM	0.78	0.75	0.75	0.78	0.65
CBAM	0.87	0.78	0.77	0.79	0.75

## Data Availability

For the detailed information of the image data, please contact the corresponding authors.
